# Antibiotic-potentiating efficacy of *Rosmarinus officinalis* L. to combat planktonic cells, biofilms, and efflux pump activities of extensively drug-resistant *Acinetobacter baumannii* clinical strains

**DOI:** 10.3389/fphar.2025.1558611

**Published:** 2025-04-08

**Authors:** Sanaz Khashei, Hossein Fazeli, Fateh Rahimi, Vajihe Karbasizadeh

**Affiliations:** ^1^ Department of Microbiology, School of Medicine, Isfahan University of Medical Sciences, Isfahan, Iran; ^2^ Department of Microbiology, Faculty of Biological Science and Technology, University of Isfahan, Isfahan, Iran

**Keywords:** *Acinetobacter baumannii*, drug resistance, biofilms, *Rosmarinus officinalis*, liquid chromatography-mass spectrometry

## Abstract

**Introduction:**

This research aimed to examine the action of commercial antibiotics against extensively drug-resistant (XDR) *A. baumannii* clinical strains when combined with *Rosmarinus officinalis* extracts.

**Methods:**

Agar well diffusion and broth microdilution were used to screen the antibacterial activity of crude ethanol extract and its fractions (hexane, intermediate, ethyl acetate, and water). The interactions between the extracts and antibiotics (gentamicin, tetracycline, cefepime, and ciprofloxacin) were evaluated by checkerboard assay. The anti-biofilm and efflux pump inhibition activities were determined by the microtiter plate method and dye accumulation assay using flow cytometry, respectively. The potential phytochemicals that contribute to the antibacterial effects of *R. officinalis* were identified using the liquid chromatography-mass spectrometry (LC–MS).

**Results:**

*R. officinalis* crude extract (CE) demonstrated the best antibacterial activity with MIC values ranging from 300 to 600 μg/mL. The combination of CE and tetracycline exhibited the highest overall synergistic effect. This combination hindered biofilm formation ranging from 21.4% to 57.31% and caused a significant increase (up to 14%) in the fluorescence intensity in 75% of the studied strains. The LC-MS analysis of CE exhibited eleven compounds in which rosmarinic acid (55.53%) was the most abundant phenolic compound followed by cirsimaritin (11.46%), and p-coumaroyl hexoside acid (10.5%).

**Discussion:**

Overall, this is the first direct report that demonstrated the efficacy of *R. officinalis* when applied with conventional antibiotics on biofilm formation and efflux pump activity in XDR *A. baumannii* clinical strains.

## 1 Introduction

*Acinetobacter baumannii* has currently emerged as the leading cause of nosocomial infections with high morbidity and mortality rates (26%–60%) due to its extensive drug resistance (Basak et al., 2016; Roy et al., 2022). Furthermore, this pathogen has now gone beyond hospitals and is being reported to cause community-acquired infections in both pediatric and adult populations. *A. baumannii* can colonize biotic and abiotic surfaces for prolonged periods in hostile conditions such as desiccation, antimicrobial therapies, and nutrient unavailability due to their ability to form complex structures called biofilms. Compared to planktonic cells, the three-dimensional architecture of biofilm provides one thousand times more tolerance to antimicrobial agents by shielding the bacterial cells from treatment with antibiotics. In addition, efflux pumps play a dual role in drug resistance either directly by extruding antibiotics from the cells or indirectly by biofilm formation (Abd El-Rahman et al., 2023). Based on the 2017 World Health Organization (WHO) Bacterial Priority Pathogens List (WHO BPPL), *A. baumannii* is considered a critical microorganism (Priority 1) for the development of novel antibiotics (Nocera et al., 2021). However, the major limitation in developing new antibiotics is financial efficiency. Also, many drugs have failed to enter the market due to their poor pharmacological attributes, leading to significant financial losses for the pharmaceutical industry. One of the potential strategies to overcome this limitation is natural-origin compounds, such as plant extracts, as antibiotic adjuvants (Romo-Castillo et al., 2023). The plant extract-antibiotic combination is an innovative alternative to prescriptive treatment protocols for expanding the antimicrobial spectrum and avoiding the emergence of resistant strains and undesirable toxic effects of antimicrobial therapy. *Rosmarinus officinalis,* commonly called rosemary, belongs to the family Lamiaceae, is an aromatic evergreen shrub distributed throughout the world and known to exhibit antitumor, antibacterial, antiviral, anti-inflammatory, diuretic, antithrombotic, antioxidant, and antidiabetic activities (Manilal et al., 2021). The biological properties of rosemary have been attributed to its phytochemical composition rich in bioactive secondary metabolites with suitable economic viability and efficacy (Mena et al., 2016). The quantitative distribution of plant secondary metabolites can vary from organ to organ or plant to plant. Liquid chromatography-mass spectrometry (LC–MS)-based approaches have particular importance in the separation and detection of highly rich biochemistry of plants due to their high selectivity, high sensitivity, and high qualitative productivity (El Sayed et al., 2020; Kaur et al., 2022). Although some studies were reported on the antibacterial activity of *R. officinalis,* the main components in this plant that contribute to its antibacterial activity remain unclear to date (Zhong et al., 2021). On the other hand, the antibacterial interactions between rosemary and many commercially available antibiotics have been overlooked. In light of this, the present study aims to evaluate the effect of crude ethanol extract of *R. officinalis* and its different fractions combined with various antibiotics on biofilm formation and efflux pump activity of extensively drug-resistant (XDR) *A. baumannii* clinical strains. Also, the detection and identification of bioactive components of *R. officinalis* were done using the LC–MS method.

## 2 Materials and methods

### 2.1 Source of bacterial strains

A total of nine biofilm-forming XDR *A. buamannii* strains belonging to nine different genotypes based on ERIC-PCR patterns, which were previously isolated from patients with burn wound infection in Isfahan, Iran, were included in this study (unpublished data). These strains were preserved at − 80°C in Tryptic Soy Broth (TSB) (Merck, Germany) supplemented with 20% glycerol. Working cultures were prepared by transferring 10 μL of frozen stock cultures to Tryptic Soy Agar (Merck, Germany) followed by incubation at 37°C for 24 h.

### 2.2 Detection of efflux pump activity using dye accumulation assay

The screening of bacterial strains for efflux pump activity was carried out by dye accumulation assay as a previously published protocol (Lu et al., 2020), with the following modifications. The bacterial cells were incubated to OD_600_ of 0.8 in TSB and collected by centrifugation for 5 min at 5,000 rpm. The cells were washed three times with phosphate-buffered saline (PBS) and resuspended with the same buffer in a final OD_600_ of 0.6. Then, the cell suspensions were stained with Rhodamine-123 (Rho-123; Sigma-Aldrich, United States) to a final concentration of 200 μg/mL (Khashei et al., 2018). After incubation in the dark for 10 min, the fluorescence was measured by a flow cytometer (BD FACSCalibur, United States) through the green channel of a fluorescence detector (FL1, 525 nm). The protonophore carbonyl cyanide 3-chlorophenylhydrazone (CCCP), which works by dissipating the proton motive force (PMF), the primary energy source for many efflux pumps (Zoaiter et al., 2023), was used as a positive control to a final concentration of 50 μg/mL. Also, unstained bacterial cells were considered as negative control. Data analysis was carried out using FlowJo v10.5.3 Software. Strains that showed lower fluorescence than the control (indicative lower level of dye accumulated in the cells) were chosen for further studies.

### 2.3 Preparation of ethanolic extract

Aerial parts of *R. officinalis* were collected in September 2023 from the campus of Isfahan University of Medical Sciences and were characterized by the Department of Plant and Animal Biology, the University of Isfahan, and a voucher number (260127) was deposited at the herbarium. The extraction process was done according to the previously described with some modifications (Moussi et al., 2015). Briefly, the dried leaves were ground to prepare powder, which was milled through a 1 mm sieve. Then, 50 g of powdered sample was macerated with 500 mL of ethanol (Merck, Germany) for 48 h at room temperature with continuous stirring. After filtration through filter paper, the extract solution was concentrated using a rotary evaporator and dried at room temperature. The dry extract was considered crude extract (CE).

### 2.4 Fractionation by liquid–liquid extraction

Further fractions were obtained by suspending 2 g of CE in distilled water (20 mL). This solution was then successively partitioned with the same volume of n-hexane and ethyl acetate (Merck, Germany) in a Pyrex 100 mL separating funnel to obtain respective solvent-solvent fractions. Also, the insoluble fraction formed between hexane and aqueous solution was collected as the intermediate fraction (INT). Each fraction was concentrated with a rotary evaporator, dried, and stored at 4°C (Moussi et al., 2015).

### 2.5 Antibacterial screening of the crude and fractional extracts

The agar well diffusion method was applied to assay the antibacterial activity of extracts against selected *A. baumannii* strains. Each extract solution was prepared at concentrations of 6.25, 12.5, 25, and 50 mg/mL by dissolving the dried extracts in 5% dimethyl sulphoxide (DMSO; Merck, Germany). Each well of the Mueller-Hinton Agar (MHA; Merck, Germany) plate was filled with 100 μL of the test substances and the plates were incubated at 37°C for 24 h. Doxycycline (Sigma-Aldrich, United States) and 5% DMSO served as positive and negative controls, respectively. All experiments were set in triplicate (Sa-Eed et al., 2023). Based on the produced zones of inhibition, the CE with the highest activity against *A. baumannii* strains was selected for further evaluation.

### 2.6 Determination of minimum inhibitory concentration (MIC)

The MIC values of CE and antibiotics alone (ciprofloxacin, tetracycline, gentamicin, and cefepime; Sigma-Aldrich, United States) were determined using the broth microdilution method. Briefly, bacterial suspension (1 × 10^6^ CFU/mL; 100 μL) was placed into each well of 96-well microtiter plates containing 100 μL twofold serial dilutions of CE and antibiotics (Nassar et al., 2021). A zero-hour reading was taken by recording the optical density (OD_600 nm_) after 30 min of incubation. Then, the microtiter plates (MTP) were incubated at 37°C for 24 h, and absorbance was measured again. The lowest concentration of antibacterial agent that prevents the growth of bacteria, compared with the controls (wells without antibacterial agents), was considered as the MIC. The assay was carried out thrice (Atta et al., 2023).

### 2.7 Assessment of interactions between antibiotics and test extract

The interactive antibacterial effect of antibiotics and CE was determined by the checkerboard microdilution technique to obtain a fractional inhibitory concentration index (FICI) (Norbury et al., 2016). In a 96-well MTP, the CE was diluted (2-fold) horizontally and antibiotics were diluted (2-fold) vertically. The total volume of the combination was 100 μL per well. Then 100 μL of the bacterial inoculum (1 × 10^6^ CFU/mL) was added to each well and incubated for 24 h at 37°C (Yang et al., 2017). The FICI was calculated by applying the formulas displayed below: FICI = FICA + FICB, where FICA was MIC of extract in combination/MIC of extract alone, and FICB was MIC of antibiotic in combination/MIC of antibiotic. The interactions were categorized as being synergistic for the FIC value of ≤0.5, partial synergistic (0.5 < FICI < 1), additive (FICI = 1), indifferent (1 ≤ FICI < 4), or antagonistic (FICI > 4.0) (Haji et al., 2022). A combination of CE and tetracycline with the highest synergistic effect was selected for further studies.

### 2.8 Antibiofilm assay

The effect of the tetracycline and CE (individually and in combination) at ½ MIC levels on the biofilm formation ability of XDR *A. baumannii* strains was tested as described by Amer et al. (2023). Briefly, MTPs were prepared as described above. After 24 h incubation at 37°C, the planktonic cells were discarded, and plates were gently washed three times with normal saline (0.9% NaCl). The remaining attached bacterial cells were fixed by adding 200 μL of ethanol (96%) to each well. After 15 min incubation at room temperature, the contents of all wells were discarded, and the adhered cells were stained using crystal violet 1% (w/v) (Sigma, Germany) for 15 min. Then, wells were washed with normal saline three times to remove excess stains. Finally, the dye was solubilized with 30% (v/v) acetic acid and the optical density of each well was measured at 570 nm using an ELISA reader (BioTek 800 TS, United States). The antibiofilm activity was expressed as biofilm inhibition (BI) percentage using the following equation (Adeyemo et al., 2022):

$$\text{BI }\left(\%\right)=\left[\left({\text{OD}}_{\text{Control}}-{\text{OD}}_{\text{Sample}}\;\right)\;/\;{\text{OD}}_{\text{Control}}\;\right]\times 100$$

Also, the biofilm formation ability of the strains was classified as follows: (1) OD ≤ ODc = negative; (2) ODc < OD ≤ 2ODc = weak; (3) 2ODc < OD ≤ 4ODc = intermediate; and (4) OD > 4ODc = strong. ODc represents the mean OD value of the wells without bacterial strains, while OD is the value of the tested strains.

### 2.9 Dye accumulation assay

The synergistic inhibitory effect of the CE in combination with tetracycline was estimated against efflux activity in biofilm-forming XDR *A. baumannii* using a dye accumulation assay. This assay was performed by growing bacterial strains in TSB until the mid-log phase. The bacterial cells were harvested by centrifugation at 5,000 rpm for 5 min and washed several times with PBS. The pellet was resuspended with the same buffer, then 0.4% glucose was added, and OD_600_ was adjusted to 0.6. The bacterial suspension was incubated for 30 min at 37°C, without or with the sub-lethal dose (½ MIC) of selected antibacterial agents (Al-Sallami et al., 2023). Then, bacterial cells were stained with Rhodamine-123 (Rho-123). After incubation in the dark for 10 min, the fluorescence was measured by a flow cytometer as described above.

### 2.10 Analysis of phytochemicals by liquid chromatography–mass spectrometry (LC-MS)

The LC-MS analysis of the CE was performed with a Waters Alliance 2695 HPLC-Micromass Quattro micro-API Mass Spectrometer. Chromatographic separation was employed on Waters Xbridge C18 5 μm, 15 cm × 4.6 mm with mobile phase, including a blend of H_2_O and 0.1% formic acid water (solvent B) and acetonitrile and 0.1% formic acid (solvent C). The column temperature was 35°C, the flow rate was set at 0.3 mL/min, and the injection volume was 10 μL (Hassan et al., 2022).

### 2.11 Statistical analysis

Results were expressed as the mean ± standard deviation for each group. Differences in quantitative measurements were assessed by Student’s t-test or one-way analysis of variance. Differences were considered statistically significant at *p* < 0.05, ∗*p* < 0.05, ∗∗*p* < 0.01, ∗∗∗*p* < 0.001, and ****P value ≤ 0.0001. All analyses were carried out with GraphPad Prism 8.0.

## 3 Results

### 3.1 Monitoring of accumulation of Rho-123 in bacterial cells

**FIGURE 1:**
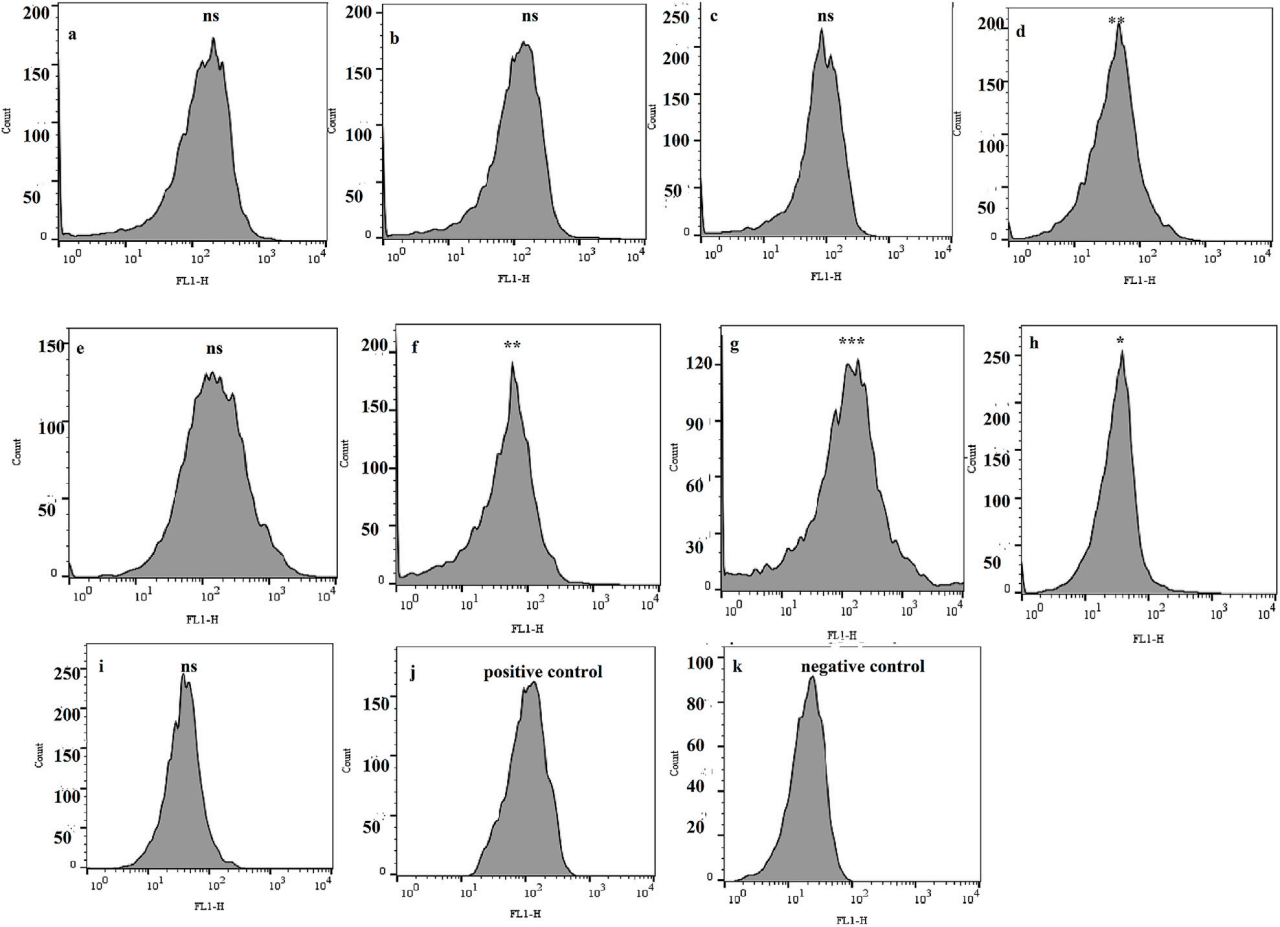
Flow cytometry plots displaying fluorescence intensities of rhodamine 123 as a marker of the efflux pump activity in biofilm-forming XDR *A. baumannii* strains. Each panel **(a–i)** corresponds to one of the nine tested bacterial strains, while panels **(j, k)** show fluorescence intensity in the positive control (treated with CCCP) and the negative control (unstained cells), respectively. The asterisks indicate significant differences compared to the positive control. ns: not significant (*P* value > 0.05), *P value < 0.05, **P value ≤0.01, and ***P value ≤0.001.

A total of nine biofilm-forming XDR *A. buamannii* strains, belonging to different genotypes isolated from burn wound-infected patients, were examined using the dye accumulation assay to monitor possible efflux pump activity. The significant lower florescent intensity was identified in four strains (44.44%) by a 1.52 (Figure 1d), 1.22 (Figure 1f), 2.39 (Figure 1g), and 1.11 (Figure 1h)-fold lower fluorescence in the respective strains compared to the positive control. The lower fluorescence intensity (lower level of R-123 accumulation) could likely be due to the action of efflux pumps. These four strains were selected for further study and were referred to as AB1, AB2, AB3, and AB4, respectively.

### 3.2 Antibacterial activity of *R. officinalis*

**FIGURE 2:**
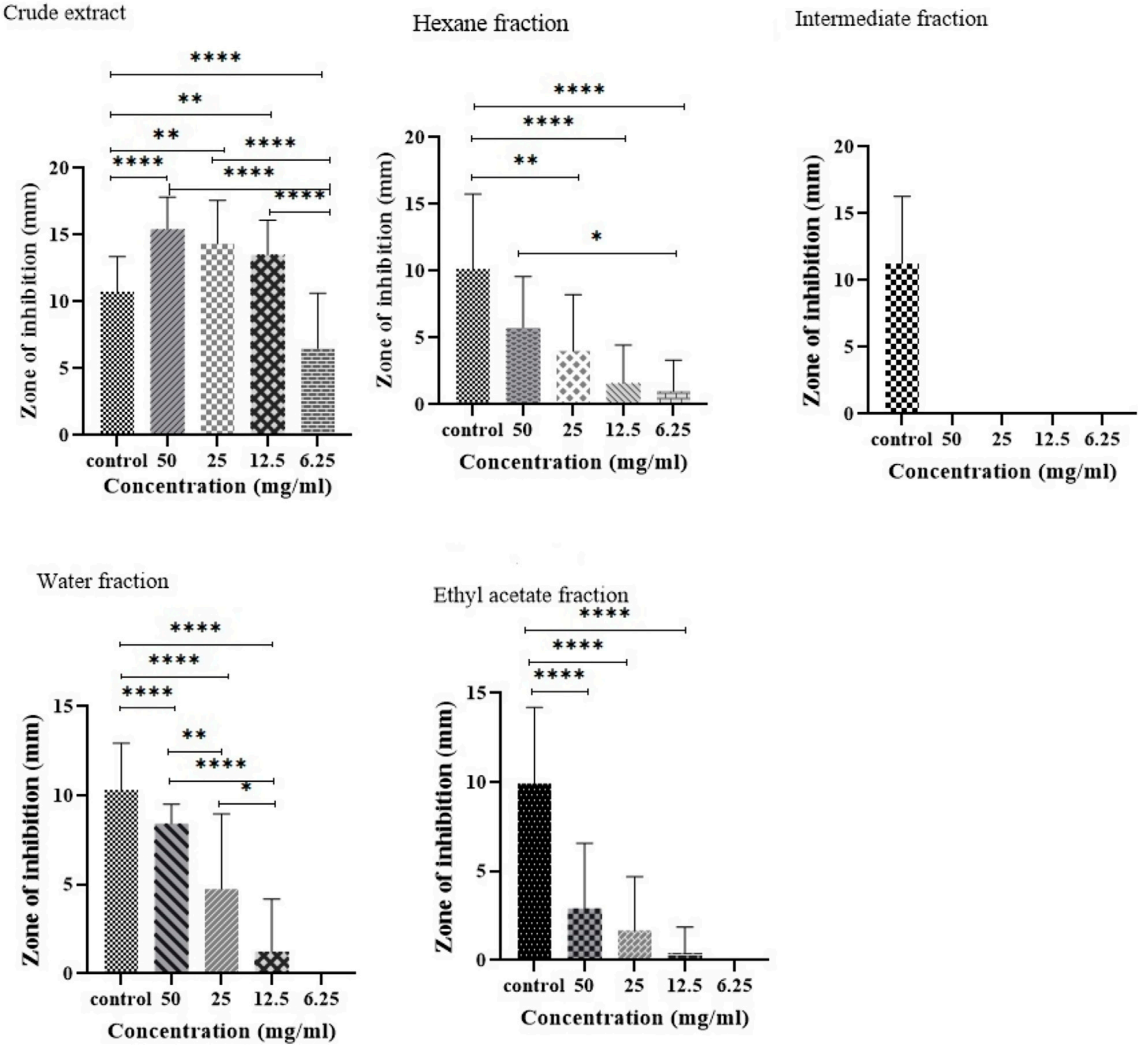
Comparison of the antibacterial activity of CE and its fractions against biofilm-forming XDR *A. baumannii* strains. *P value < 0.05, **P value ≤ 0.01, and ****P value ≤ 0.0001.

The antibacterial activity was evaluated against biofilm-forming XDR *A. baumannii* strains. It was found that the antibacterial activity of plant extract and its fractions differed based on the type of solvent used in the extraction process (Figure 2). Compared to the positive control, a significant dose-dependent inhibition of bacterial growth by CE was observed. The CE showed remarkable antibacterial activity with a wider inhibition zone (15.4 mm) at a concentration of 50 mg/mL and even at lower concentrations of 25 mg/mL (14.3 mm) and 12.5 mg/mL (13.5 mm). In contrast, the intermediate fraction had no inhibitory effect on the growth of bacteria.

### 3.3 Minimum inhibitory concentration (MIC)

The results of MIC determination for the CE and each of the four tested antibiotics are shown in Table 1. CE was found to have potent antimicrobial activity against biofilm-forming XDR *A. baumannii* strains, with a mean MIC ranging from 300 to 600 μg/mL. The MIC values for tetracycline and gentamycin ranged from 32 to 64 μg/mL, while tested strains exhibited ciprofloxacin and cefepime MIC values of 64–128 and 128–256 μg/mL, respectively. Based on these results, all bacterial strains showed MICs in the antibiotic resistance range.

**TABLE 1:** MIC values for tested antibacterial agents measured by broth microdilution method against biofilm-forming XDR *A. baumannii* strains.

Bacterial strain	MIC values of antibacterial compounds (μg/mL)				
	CE	TE	CN	FEP	CIP
AB1	300	64	32	128	128
AB2	300	64	64	256	64
AB3	600	64	64	128	128
AB4	300	32	64	256	64

Abbreviations are CE, crude extract; CN, gentamicin; TE, tetracycline; FEP, cefepime; and CIP, ciprofloxacin.

### 3.4 Interaction of CE with different antimicrobials

We chose four antimicrobial classes including, fluoroquinolones (ciprofloxacin), aminoglycosides (gentamicin), tetracyclines (tetracycline), and cephalosporins (cefepime), to test their potential synergistic effects CE. With the addition of CE, a significant decrease in MIC was observed in the case of gentamicin (*P* value = 0.024), tetracycline (*P* value = 0.0012), and cefepime (*P* value = 0.0114). The combination of CE and tetracycline exhibited the highest overall synergistic effect, with an inhibitory concentration up to 16 times lower than the inhibitory concentration of tetracycline monotherapy (FIC values ranging from 0.3125 to 0.375) (Table 2). No antagonistic activity was observed with any of the combinations. The combination of CE with gentamicin exhibited a partial synergistic effect in 25% of studied bacterial strains (FIC value = 0.75) (Table 3), while the combination of CE with ciprofloxacin (Table 4) and cefepime (Table 5) exhibited indifferent effects when combined with CE.

**TABLE 2:** MICs and FIC indices of tetracycline in combination with CE extract against biofilm-forming XDR *A. baumannii* strains.

Bacterial strain	MIC for combination of two antibacterial compounds (μg/mL)		FIC of individual compounds		The sum of FIC of two compounds in the combination	Interaction between antibacterial agents
	CE	TE	CE	TE		
AB1	75	4	0.25	0.0625	0.3125	Synergistic
AB2	75	4	0.25	0.0625	0.3125	Synergistic
AB3	75	16	0.125	0.25	0.375	Synergistic
AB4	75	4	0.25	0.125	0.375	Synergistic

Abbreviations are TE, tetracycline; and CE, crude extract.

**TABLE 3:** MICs and FIC indices of gentamicin in combination with CE against biofilm-forming XDR *A. baumannii* strains.

Bacterial strain	MIC for combination of two antibacterial compounds (μg/mL)		FIC of individual compounds		The sum of FIC of two compounds in the combination	Interaction between antibacterial agents
	CE	CN	CE	CN		
AB1	150	32	0.5	1	1.5	Indifferent
AB2	150	32	0.5	0.5	1	Indifferent
AB3	300	32	0.5	0.5	1	Indifferent
AB4	75	32	0.25	0.5	0.75	Partial synergistic

Abbreviations are CN, gentamicin; and CE, crude extract.

**TABLE 4:** MICs and FIC indices of ciprofloxacin in combination with CE against biofilm-forming XDR *A. baumannii* strains.

Bacterial strain	MIC for combination of two antibacterial compounds (μg/mL)		FIC of individual compounds		The sum of FIC of two compounds in the combination	Interaction between antibacterial agents
	CE	CIP	CE	CIP		
AB1	300	64	1	0.5	1.5	Indifferent
AB2	300	64	1	1	2	Indifferent
AB3	300	64	0.5	0.5	1	Indifferent
AB4	150	64	0.5	1	1.5	Indifferent

Abbreviations are CIP, ciprofloxacin; and CE, crude extract.

**TABLE 5:** MICs and FIC indices of cefepime in combination with CE against biofilm-forming XDR *A. baumannii* strains.

Bacterial strain	MIC for combination of two antibacterial compounds (μg/mL)		FIC of individual compounds		The sum of FIC of two compounds in the combination	Interaction between antibacterial agents
	CE	FEP	CE	FEP		
AB1	150	64	0.5	0.5	1	Indifferent
AB2	300	64	1	0.25	1.25	Indifferent
AB3	600	32	1	0.25	1.25	Indifferent
AB4	300	64	1	0.25	1.25	Indifferent

Abbreviations are FEP, cefepime; and CE, crude extract.

### 3.5 Anti-biofilm activities of *R. officinalis*/tetracycline combination

The findings of this study, using the quantitative microtiter plate method for the determination of biofilm formation ability, showed that all four tested *A. Baumannii* strains (100%) produced biofilm. Among these four biofilm-producing strains, 25% produced moderate biofilms and 75% formed strong biofilms. The anti-biofilm activity of rosemary CE alone and in combination with tetracycline at ½ MIC level against these strains was summarized in Table 6. The combination of CE with tetracycline achieved the highest inhibition of biofilm formation against various examined strains (BI (%) varied from 21.4% to 57.31%), followed by CE (BI (%) ranged from 12.5% to 54.34%).

**TABLE 6:** Biofilm inhibition (BI) percentage of tetracycline in combination with CE against XDR *A. baumannii* strains. Data represent the mean of three biological replicates ±standard deviation (Yilmaz et al., 2014).

Tested strains (biofilm formation ability)	CE	TE	CE/TE
AB1 (S)	54.34% ± 0.03 (****)	29.69% ± 0.08 (***)	57.31% ± 0.16 (****)
AB2 (M)	12.25% ± 0.03	3.97% ± 0.07	29.38% ± 0.07 (*)
AB3 (S)	16.66% ± 0.32	8.98% ± 0.18	21.40% ± 0.369
AB4 (S)	13.5% ± 0.15	5.81% ± 0.35	36.21% ± 0.56 (**)

The inhibition (%) was calculated from biofilm control (untreated cell). CE, crude extract; TE, tetracycline; S, Strong biofilm producer; M, Medium biofilm producer. Statistical analysis was performed using one-way ANOVA, and Tukey’s test (*P* < 0.05). The significant differences between the control and each treatment are represented by stars (*P value < 0.05, **P value ≤0.01, ***P value ≤0.001, ****P value ≤0.0001).

### 3.6 Fluorescent dye accumulation reduced by *R. officinalis*/tetracycline combination

**FIGURE 3:**
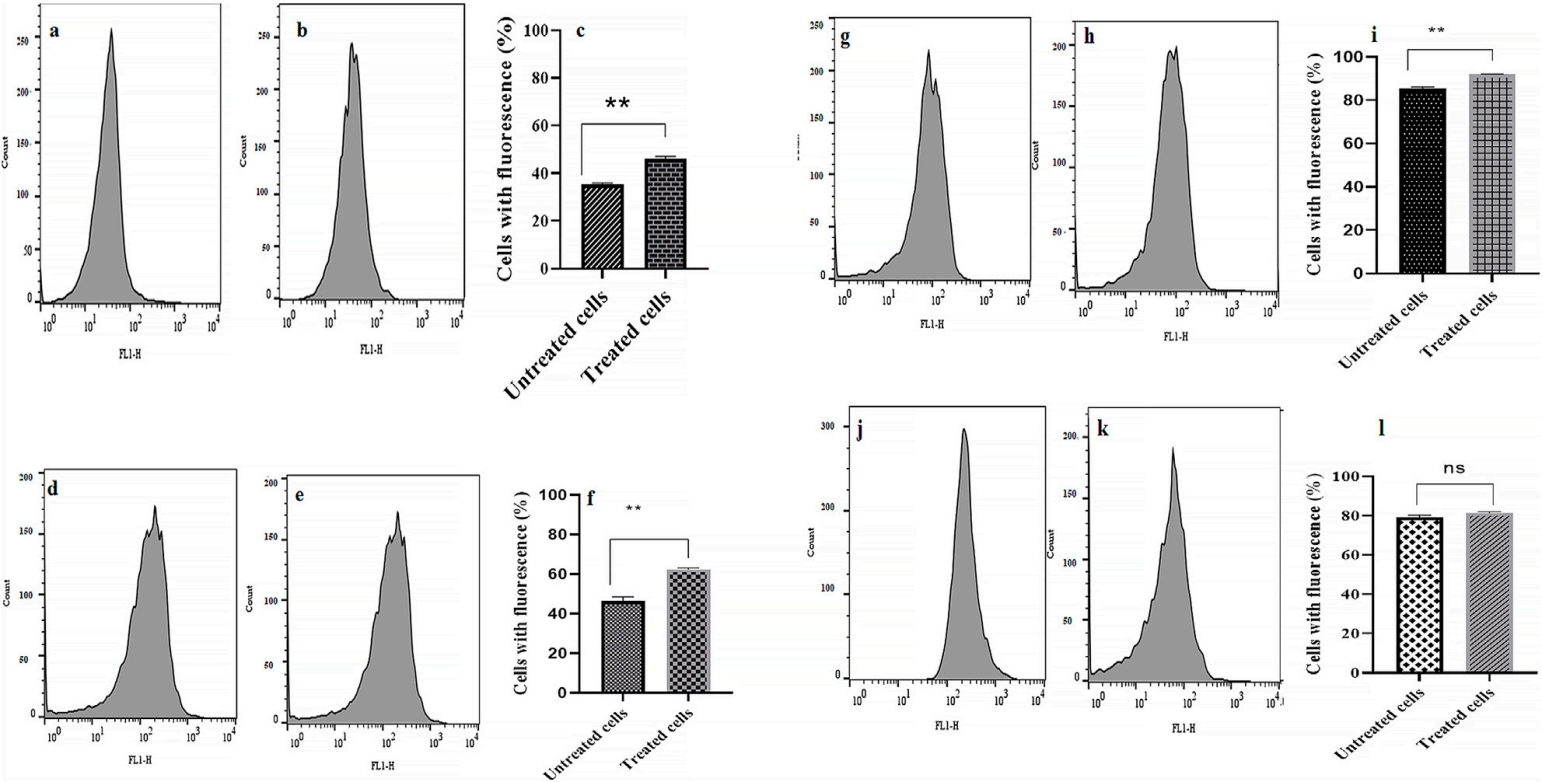
Evaluation of dye accumulation in bacterial cells in the absence and presence of treatment with CE/tetracycline combination. Panels **(a, d, g, and j)** show flow cytometry plots indicating fluorescence intensity in the absence of treatment, panels **(b, e, h, and k)** show fluorescence intensity in the presence of treatment, and panels **(c, f, i, and l)** present comparison graphs of fluorescence intensity (dye accumulation) in the absence and presence of treatment, in strains AB1, AB2, AB3, and AB4, respectively. ns: not significant (*P* value > 0.05) and **P value ≤0.01.

Compared to the control group (0 MIC), the addition of CE in combination with tetracycline caused a significant increase (up to 14%) in the fluorescence intensity (indicates a higher level of R-123 accumulation) in 75% of the studied bacterial strains (Figure 3).

### 3.7 Phytochemical analysis of CE by LC-MS

**FIGURE 4:**
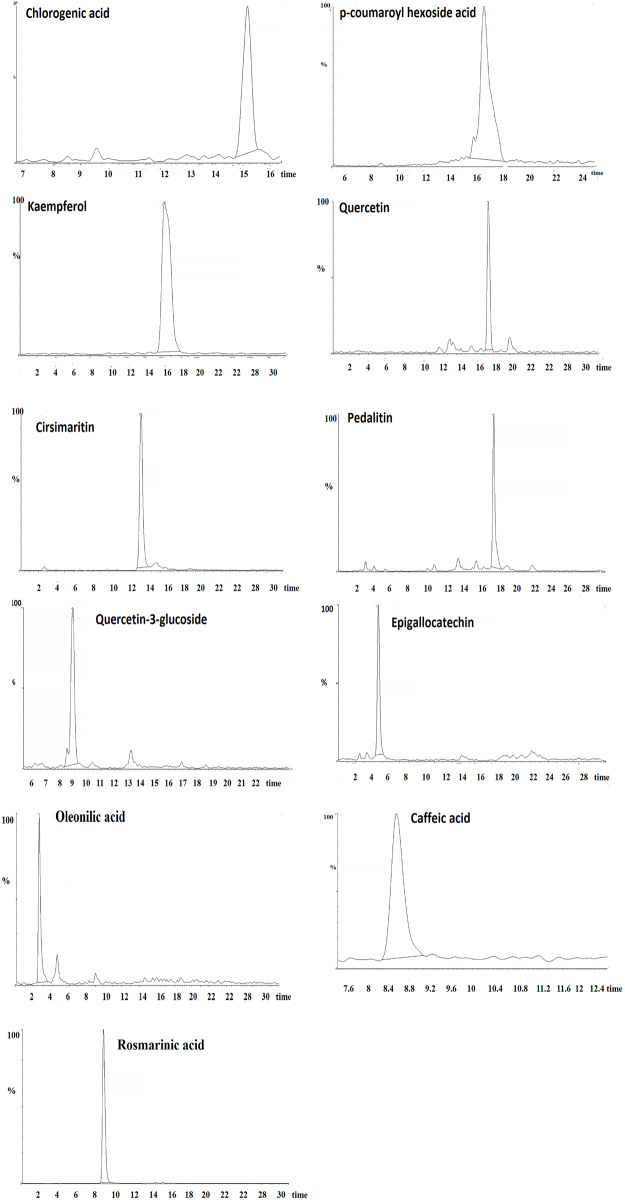
LC-MS chromatogram and chemical structure of identified bioactive compounds in CE.

According to the LC-MS analysis, eleven peaks were tentatively characterized by comparing their molecular weight and mass fragmentation pattern in their LC-MS spectra with published literature data. These identified constituents included oleanolic acid (retention time (RT): 2.59 min), epigallocatechin (RT: 4.57 min), caffeic acid (RT: 8.59 min), quercetin-3-glucoside (RT: 8.87 min), rosmarinic acid (RT: 9.15 min), cirsimaritin (RT: 13.76 min), chlorogenic acid (RT: 15.26 min), kaempferol (RT: 16.33 min), p-coumaroyl hexoside acid (RT: 16.63 min), quercetin (RT: 17.61 min), and pedalitin (RT: 17.82 min) (Figure 4). Also, by calculating the area under the peak, the relative amount of each compound was measured as a percentage of the total area of the peaks in the chromatogram. In this regard, CE was dominated by rosmarinic acid (55.53%), followed by cirsimaritin (11.46%), and p-coumaroyl hexoside acid (10.5%) (Table 7).

**TABLE 7:** Compounds identified in the CE.

Peak no.	Identification	Formula	[M-H]-(m/z)	Structure class	Peak area (%)
1	Oleanolic acid	C_30_H_48_O_3_	455.7	Triterpenoid	1.59
2	Epigallocatechin	C_15_H_14_O_7_	305.74	Flavonoid	4.03
3	Caffeic acid	C_9_H_8_O_4_	179.533	Phenolic acid	1.37
4	Quercetin-3-glucoside	C_21_H_20_O_12_	461.821	Flavonoid	2.03
5	Rosmarinic acid	C_18_H_16_O_8_	359.679	Phenolic acid	55.53
6	Cirsimaritin	C_17_H_14_O_6_	313.747	Flavonoid	11.46
7	Chlorogenic acid	C_16_H_18_O_9_	353.751	Phenolic acid	1.59
8	Kaempferol	C_15_H_10_O_6_	285.924	Flavonoid	6.64
9	p-Coumaroyl hexoside acid	C_15_H_18_O_8_	325.784	Flavonoid	10.5
10	Quercetin	C_15_H_10_O_7_	301.865	Flavonoid	2.03
11	Pedalitin	C_16_H_12_O_7_	315.842	Flavonoid	3.54

## 4 Discussion

In the absence of newly emerging antibacterial agents that are superior to currently available options against XDR *A. baumannii* infections, one approach that has been explored is combination therapy to improve outcomes (Spellberg and Bonomo, 2015). The antibacterial activity of *R. officinalis* against multiple pathogens was demonstrated in previous studies (Del Campo et al., 2000; Manilal et al., 2021; Günther et al., 2022). To the best of our knowledge, colistin is the only antibiotic examined in combination with *R. officinalis* against XDR *A. baumannii* clinical isolates previously (Kafa et al., 2022). Thus, the antibacterial synergism activity of rosemary with many of the conventional antibiotics has been overlooked. On the other hand, treatment regimens including colistin should be prescribed carefully because the clinical use of colistin is accompanied by a narrow therapeutic window and certain side effects such as nephrotoxicity and neurotoxicity (Samarkos et al., 2022). In this view, the current study evaluates the effects of *R. officinalis* extract combined with various antibiotics from four antimicrobial classes on biofilm formation and efflux pump activity of XDR *A. baumannii* clinical strains. Our results showed that CE has the greatest antibacterial activity and the fractionation process reduced its antibacterial activity. This indicates that the active compounds might be more concentrated in the CE and more diluted in its fractions. Also, a polarity-dependent decrease in antibacterial properties was observed among different fractions achieved from solvents with various polarities (water, ethyl acetate, and hexane as polar, semi-polar, and non-polar solvents, respectively). This suggests that the aerial parts of *R. officinalis* contain several antibacterial compounds with different polarities and the polarity of the solvent greatly influences its antimicrobial activity. Consistent with our findings, Saxena et al. reported that increased polarity aids the entry of a compound into Gram-negative bacteria. The higher polarity of compounds targeting Gram-negative bacteria corresponds to the fact that the compounds are looking for their route through the hydrophilic porin channels (Saxena et al., 2023). There are numerous criteria for the classification of the antimicrobial activity of plant extracts. According to Simoes et al., an extract is said to be antimicrobial when MIC ranges from 100 to 1,000 μg/mL (Simoes et al., 2009), while according to Aligiannis et al., the antimicrobial activity of an extract is considered strong when MIC < 500 μg/mL, moderate when MIC values ranged between 500 μg/mL to 1,500 μg/mL, and low when greater than 1,500 μg/mL (Aligiannis et al., 2001). In this regard, the ethanolic crude extract of *R. officinalis* demonstrated remarkable antimicrobial activity against *A. baumannii* with a MIC of 300–600 μg/mL in the current study. The MIC values of the ethanolic extract and essential oil of rosemary were reported previously to be 1–2 mg/mL and 5–20 μg/mL against *A. baumannii,* respectively (Assis et al., 2018; Kafa et al., 2022). These differences may be due to the variation of the chemical composition of plant extracts in response to seasonal variation, culture conditions, the clime, and even the extraction method which can affect their antibacterial activity (5).

The combination of CE with different antibiotics has shown various effects on XDR *A. baumannii* strains. In our study, the *R. officinalis* ⁄ tetracycline combination displayed the most favorable synergistic pattern, and the MIC of tetracycline reached the sensitivity threshold (MIC ≤ 4) in 75% of tested strains when applied in combination with rosemary. Also, we observed that the *R. officinalis* ⁄ gentamicin combination only produced a partial synergistic effect in 25% of tested strains and indifferent interaction in other strains. Based on our findings, the combination of CE with ciprofloxacin and cefepime showed indifferent interactions. Although the MIC values of these antibiotics were reduced by 2 to 4-fold, the strains remained resistant to these antibiotics. It is generally agreed that a combination of multiple antimicrobial agents can yield various effects depending on the chemical nature of their components, the intermolecular interactions, and the concentration of the compounds (Vaou et al., 2022). Therefore, this study can serve as a guide for future research on the use of combinations having promising synergistic profiles with fewer side effects and a more positive contribution.

The exact mechanism by which natural antimicrobial agents decrease bacterial resistance to antibiotics is unknown. Previous studies suggest that these compounds may simplify drug penetration through the outer layers of bacterial cell walls, block the inhibitory effect of protective enzymes, and interfere with the metabolic targets of the antibiotic (Górniak et al., 2019). In addition, they often contain compounds that increase the solubility, absorption, distribution, or metabolism of active constituents of antibiotics (Vaou et al., 2022). Based on our results, *R. officinal* might act as a resistance breaker that can restore the activity of tetracycline against *A. baumannii* strains, in case of the biofilm- and efflux-mediated antimicrobial resistance mechanisms.

Based on the LC-MS profile, the potential bioactive metabolites that contribute to the antimicrobial effects of CE were similar to those of other studies in the literature (Borrás-Linares et al., 2014; Christopoulou et al., 2021), except for pedalitin. The antifungal effects of pedalitin (5,6,3′,4′-tetrahydroxy-7-methoxyflavone) obtained from *Pterogyne nitens* have been demonstrated previously against several strains of *Candida albicans* and *Cryptococcus* spp. (Sangalli-Leite et al., 2016). Shoba et al. reported that pedalitin is responsible for the inhibitory activity of *Pedalium murex* against *Proteus mirabilis* (Ramadevi et al., 2020). Another study conducted by Lin et al. showed that pedalitin isolated from *Rabdosia serra* has considerable anti-melanogenesis and anti-diabetic effects by inhibition of tyrosinase and α-glucosidase enzymes, respectively (Lin et al., 2011). Thus, this is the first study reporting the crude ethanol extract of rosemary as a novel natural source for the isolation of pedelitin.

Plant bioactive secondary metabolites include flavonoids, terpenoids, and phenolic acids, which were represented to have significant antimicrobial effects (Bouyahya et al., 2022). The antibiofilm activity of flavonoids and phenolic acids such as quercetin, epigallocatechin, rosmarinic acid, and kaempferol as bioactive active secondary metabolites derived from medicinal plants against various pathogenic bacteria has been demonstrated previously. These molecules suppressed the quorum sensing process as a main regulatory system in bacterial biofilm formation (Slobodníková et al., 2016). Also, the mechanism of antibiofilm action of terpenoids can be attributed to their anti-cell-adhesion properties (Lahiri et al., 2019). However, considering the method used in this study to assess the effect of the *R. officinal* extract on biofilm formation, it is possible that the extract does not directly inhibit biofilm formation but rather affects cell growth, which in turn could impact the cells’ ability to form biofilms. On the other hand, plant phenolic compounds act as protonophores which interfering with ATP synthesis and proton gradients, leading to efflux pump inhibition activity (Zhang et al., 2023). Thus, the potential of *R. officinalis* ethanolic crude extract for inhibiting efflux pump activity and biofilm formation ability in XDR-*A. baumannii* strains could be due to the presence of these bioactive compounds. Also, the whole extract derived from rosemary may have greater antibacterial potential than their single bioactive ingredients due to the synergism between their molecules and the diversity of the mechanisms of action of its compounds.

## 5 Conclusion

This is the first direct report that provides evidence supporting the potential of *R*. *officinalis* for enhancing the efficacy of commercially available antibiotics in different classes including, fluoroquinolones, aminoglycosides, tetracyclines, and cephalosporins against XDR *A. baumannii* clinical strains. The combination of CE with tetracycline exhibited significant efficacy for reversing efflux pump activity and inhibiting the biofilm formation ability of XDR *A. baumannii* strains. Furthermore, the identification of various bioactive compounds in CE underscores its potential in drug development targeting diverse health disorders.

Collectively, this work presents an innovative therapeutic approach that can be developed into clinically antibacterial agents to address the growing threat of biofilm-forming XDR *A. baumannii* and other priority pathogens. However, the study acknowledges certain limitations. Standardization challenges in studying plant-based antimicrobials and the dynamic nature of protein-ligand interactions can affect the robustness and applicability of the suggested antibacterial agents in the present study. Also, future investigations should evaluate the efficacy of the suggested antibacterial agents in the present study in animal infection models.

## Data Availability

The original contributions presented in the study are publicly available. This data can be found here: https://doi.org/10.6084/m9.figshare.28737293.
